# International collaborative research, systems leadership and education: reflections from academic biomedical researchers in Africa

**DOI:** 10.3389/feduc.2023.1217066

**Published:** 2024-01-08

**Authors:** Elizabeth S. Rose, Halima Bello-Manga, Theodore Boafor, Muhammad Asaduzzaman

**Affiliations:** 1 Vanderbilt Institute for Global Health, Vanderbilt University Medical Center, Nashville, TN, United States; 2 Department of Hematology and Blood Transfusion, Barau Dikko Teaching Hospital/Kaduna State University, Kaduna, Nigeria; 3 Department of Obstetrics and Gynecology, University of Ghana Medical School, Accra, Ghana; 4 Department of Obstetrics and Gynecology, Korle-Bu Teaching Hospital, Accra, Ghana; 5 Department of Community Medicine and Global Health, Institute of Health and Society, Faculty of Medicine, University of Oslo, Oslo, Norway

**Keywords:** global health, systems leadership, research colonialism, power imbalance, capacity building, health system research in Africa and global south, grant management, Interpretative Phenomenological Analysis (IPA)

## Abstract

**Scope:**

Academic biomedical researchers and educators in African countries navigate complex local, national, and international systems to conduct grant-funded research. To secure funding, collaboration with researchers from high-income countries is often necessary. Existing literature highlights that these global health initiatives are commonly fraught with unequal power dynamics and lead by the foreign partners. Despite these inequalities, African faculty can benefit from these collaborations, fostering the development of their labs and careers. This study delves into reflections on lived experiences from academic biomedical researchers in Africa to better understand the impact of foreign collaborations.

**Methods:**

We designed a qualitative study using the Interpretative Phenomenological Analysis (IPA) method and used Self-determination and Complex Systems Leadership theories to frame this study. Ten academic biomedical researchers in Africa consented to join this study. The participants submitted a four-week series of reflective journals through an online data management platform. Subsequently, IPA methods were employed to analyze the collected journals.

**Results:**

Participants’ reflections yielded six thematic key findings, encompassing their experiences in international collaborative research. The findings included: foreign dominance within the international macrosystem; resource challenges in their local microsystems; mesosystem dependency from collaborations; scholarly identity within research relationships; collaborative leadership; and the impact of the foreign perspective. From these findings, three implications were drawn suggesting that participants were (1) motivated by scholarly identity found in collaborations; (2) depended on collaborations that were colonialist but beneficial; and (3) created change through leadership at the microsystem level.

**Implications:**

Foreign collaborators and funders in global health education and research should critically consider how implications of this study relate to their collaborative work. Based on our analysis, recommendations for foreign collaborators and funders include prioritizing local leadership and perspectives in education initiatives and research grants; reviewing and leveraging collective leadership; engaging in bidirectional training, and mentoring opportunities; participating in power assessments; and removing publication barriers for researchers in Africa (and low-and middle-income countries). Insights from this study could impact global health research and education in multiple ways including new initiatives in mentorship and training in international collaborations along with increased awareness and correction of colonialism within these collaborations.

## 1 Introduction

Academic biomedical research in Africa, over the past three decades, has led to interventions and innovations that helped curb the epidemics of infectious diseases, including human immunodeficiency virus (HIV), tuberculosis, and malaria (Chu et al., 2014). Although the African region continues to have the highest prevalence of infectious diseases (Institute for Health Metrics and Evaluation, 2016), increased life expectancy has ushered in heightened rates of non-communicable diseases such as heart disease, diabetes, and cancers that generally present later in life (Airhihenbuwa et al., 2016). This double burden of infectious and non-communicable diseases presents a pressing need for further research capacity that will produce pioneering and sustainable healthcare solutions.

In response to calls to action for addressing this imbalance between disease burden and research, there has been a steady increase in interest to conduct research in Africa among researchers outside the continent, mostly from high-income countries in North America and Europe. However, such externally led research initiatives have not been without controversy, challenges, and ethical issues (Parker and Kingori, 2016). These challenges include unequal distribution of resources and work, power imbalances, misalignment with local research and disease priorities, and lack of local leadership (Parker and Kingori, 2016; Mlotshwa et al., 2017; Matenga et al., 2019). This phenomenon has been referred to by many terms but most commonly as scientific or research colonialism (Galtung, 1967; Jentsch and Pilley, 2003; Fennell and Arnot, 2008; Boshoff, 2009).

In research colonialism, foreign research partners propose the research agenda, which may not align with local priorities, and assume most of the credit for the research (Kok et al., 2017). The way in which research colonialism affects the researchers in African countries is to disempower and minimize local researchers in academia (Schneider and Maleka, 2018; Matenga et al., 2019). To mitigate research colonialism, many fundamental issues need to be addressed among which structural and funding systems are two major challenges African researchers encounter in leading health research (Kumwenda et al., 2017; Harsh et al., 2018; Nordling, 2018). Conducting research is often a complex interaction of institutional support, national infrastructure, international collaborations, and funding (Tulloch-Reid et al., 2018; Ezeh and Lu, 2019; Storeng et al., 2019). These issues at the micro-, meso-, and macro-system levels are often beyond the researchers’ control but impact their research productivity and agency. A majority of global health research is controlled by bodies outside of the researched community (Shrum, 2005; Harsh et al., 2018) and power imbalances in international partnerships will exist as long as non-local researchers or institutions receive research funding as grant (Matenga et al., 2019).

In this context, training programs to increase academic biomedical capacity in low-and middle-income countries (LMICs), including those in Africa, have been proposed as a strategy to strengthen researchers’ knowledge and skills as well as to reduce research colonialism (Kilmarx et al., 2018). Despite an abundance of training programs, there has been a lack of substantial reduction of research colonialism and negligible progress in research productivity among local researchers (Ezeh and Lu, 2019). Considering the noted barriers, building local administrative and grantsmanship capacity as well as teams of researchers and leaders have been proposed recently as a solution (Tulloch-Reid et al., 2018). However, there is still a scarcity in the academic literature to support this potential solution.

Apart from training needs, advocates for research equity vouch for local researchers’ strong leadership of research projects (Airhihenbuwa et al., 2016). There has been an increased awareness of the need for research collaborations in Africa to be led by those within the community or country of focus. Airhihenbuwa et al. (2016) have encouraged researchers in LMICs to “claim your space” (p. 21S) in the research landscape. Frustratingly, only 10% of worldwide health research and development funding is spent in LMICs where over 90% of the world’s preventable diseases occur, a paradox known as the “10/90 gap” (Kilama, 2009). This inequality might explain why local researchers lack substantial roles in research leadership and representation in authorship.

After surveying African researchers, Ager and Zarowsky (2015) concluded that these researchers had difficulties establishing authority over research and funding priorities since much of the research funding in Africa originated in high-income countries. Research methods and priorities typically have aligned with scientific and cultural traditions from the funder country, rather than where the research is conducted, which has led to research projects that have neglected local health needs (Jentsch and Pilley, 2003; Vasquez et al., 2013; Munung et al., 2017; Matenga et al., 2019). Collaborations between local researchers and foreign partners should include a shared research agenda driven and led by the local researcher (Seo et al., 2020). Local coordination and leadership of projects, priority setting, and authorship as well as a plan for long-term sustainable collaboration have been shown to contribute to effective international partnerships (Chu et al., 2014).

To plan for effective international research collaboration, a comprehensive understanding of the institutional, national, and international challenges encountered by African researchers in grant development, leadership, and their scholarly identity is crucial. In pursuit of this understanding, we conducted an interpretive phenomenological analysis (IPA) study among African academic biomedical researchers who participated in a training program focused on grant writing and research ethics at a university in the United States of America (USA). We aimed to explore their reflections of their lived experiences in writing and leading research grants with foreign collaborators. The research questions guiding our study were: How do African academic biomedical researchers, navigating the constraints of a culture of research colonialism, interpret their international grant collaborations at the individual, organizational, and international levels? How have these experiences influenced their individual scholarly identity?

## 2 Methods

### 2.1 Theoretical frameworks

In this study, two major theories were employed as analytical frameworks: Self-determination Theory and Complex Systems Leadership Theory.

Self-determination theory provided a framework to examine intrinsic and extrinsic motivations that guided participants’ perseverance and behaviors. According to theorists, growth is spurred by positive motivations, but stagnation may occur if psychological needs, which include autonomy, competence, and relatedness, are not met (Ryan and Deci, 2000). We proposed that these three elements would be integral parts of participants’ experiences: autonomy in guiding their academic research; competence from skill building in a training program (from which the participant pool was selected); and relatedness in collaborations. However, due to challenges described in the literature, we hypothesized that participants would experience these elements to varying degrees, which according to the theory, could hinder motivation, perseverance, and growth.

Complex Systems Leadership Theory served as a framework to understand how networks of individuals within systems interact to overcome challenges (Hazy et al., 2007). Organizations, conceptualized as living organisms, consistently encounter internal and external challenges and adapt to survive (Weberg, 2012). When faced with such challenges, leadership emerges as individuals across organizational levels and boundaries interact and link together to discover adaptive solutions (Hazy et al., 2007). These interactions lead to the emergence of new, superior behavior patterns, and successful, adaptive behaviors are internalized. Informal leaders often arise within these interactions to guide the group toward new norms. This framework was chosen to shed light on how participants potentially assume leadership roles within the intricate, complex networks of institutions and partners involved in international collaborative research.

### 2.2 Study design

To explore the research questions, we used IPA methods to collected and analyze qualitative data. IPA provides tools for engaging deeply with participants about a particular experience (Smith et al., 2009). In IPA methods, after collecting participants’ experiences of a phenomenon, researchers interpret participants’ perceptions (Smith and Osborn, 2008) to understand how they make sense of the experience. We gathered participants’ perspectives on collaborative research experiences through one-on-one engagement, facilitated by written journal entries.

Fifty African academic biomedical researchers from Ghana, Mozambique, Nigeria, Tanzania, and Zambia who completed a grant writing training program at Vanderbilt University in the USA were invited to participate. This program was chosen because it is the only known USA-based program that trains academic biomedical researchers from LMICs in grant writing so they can establish their authority and leadership as principal investigators on internationally funded research grants (Cassell et al., 2022). Most of the program’s trainees are in Africa and collaborate with researchers at Vanderbilt. Inclusion criteria for study participants were: From an African country; Currently or previously engaged in foreign grant-supported academic biomedical research collaborations for at least 2 years; Hold a degree in a health-related field (e.g., MBBS, MBChB, MD, PhD); Be able to use a web-based research instrument; Be able to write responses in English; and Be an alum of the aforementioned training program. Ten researchers consented to participate and submitted a four-week series of reflective journals.

### 2.3 Data collection

We used a constructivist epistemology perspective, which views reality as constructed by individuals. Since people’s perceptions of an event or item provide its reality, participant journals were selected as the data collection tool to capture individual experiences. We selected this format to increase distance between the participants and research team in order to minimize power imbalances and influence (Morrell-Scott, 2018). Ten individuals consented to participate and submitted journal entries, which is the highest of the general sample range (four to ten participants) suggested for IPA studies (Smith et al., 2009). IPA research is idiographic and focuses on detailed examinations of individuals’ lived experiences. Our sample size provided the ability to gather rich descriptions of participants’ experiences and conduct in-depth examinations of those experiences.

We obtained ethics (IRB) approval for this study prior to contacting potential participants. We emailed individuals a link to a consent letter with information about the study, including assurance of confidentiality in their responses. After providing consent to participate, they received a series of four reflective journal prompts, each with a main question and sub-questions that were informed by the study’s research questions. We instructed participants to write one journal entry of 500–1,500 words for each of the four prompts. They could respond to one each week or respond at more frequent intervals as their time permitted. This flexible format provided participants an opportunity to guide the research, which is an element of IPA, as well as sufficient time to reflect on topics. After submitting their entries, participants were asked to complete a demographic survey that queried on their country, gender, years in collaborative research, and areas of research expertise. All information was submitted electronically and anonymously using REDCap (Harris et al., 2009, 2019), a secure online data management platform.

### 2.4 Data analysis

For each participant’s set of journals, one researcher conducted multiple readings, interpreting, and coding of the writings in NVivo 12 software (QSR International, Melbourne, Australia) before proceeding to the next participant’s journal set. The coding process involved applying concepts from the theoretical frameworks and relevant academic literature on the topic, as described earlier. Following close readings and interpretation, we organized codes into themes, derived from the research questions and frameworks, as well as areas of convergence and divergence in experiences across participants.

## 3 Results and findings

Participants were located in four African countries, Ghana, Mozambique, Nigeria, and Zambia (however, one participant did not disclose their country, so a fifth country may have been represented) (Table 1). There were three females, six males, and one individual who did not disclose their gender. Participants reported being involved in collaborative research with foreign partners for an average of 6 years (range: 2–15; mode: 5). Six thematic findings emerged from their reflections: foreign dominance within the international macrosystems, resource challenges in their local and microsystems, dependency on collaborations creating a mesosystem, their scholarly identity within research relationships, collaborative leadership in local teams, and the impact of the foreign perspective (Table 2).

### 3.1 Finding 1—macrosystem: foreign dominance

Internationally, participants encountered power imbalances and foreign dominance throughout the grant process. In the participants’ reflections, it was evident that power inequities were present from the beginning of the grant process. Grant topics often came from foreign funders and collaborators. Topics were often not aligned with local needs, prompting participants to adjust their research areas. These power dynamics continued to be described in grant writing and submission, foreign leadership dominance during the grant, and publishing results in international journals. However, participants also reflected on mutual benefits in these relationships and wrote about the importance of collaboration. With such juxtaposition, it was often hard to interpret underlying meaning in participants’ experiences.

Participants described power inequities, beginning with research topics and collaborations requested by grant funders but not aligned with local needs. **Participant 9** explained how their local research topics were influenced by foreign funding, and how s/he and other researchers had to tailor their research areas to fit that funding agenda. S/He described,

“The research agenda here, like in many other African countries, is guided by external funders. Thus, one has to keep responding to external calls for proposals. The last decade was largely taken by HIV/AIDS research. There was minimal interest in non-HIV research. Most of us had to find some niche within the HIV arena to keep relevant. This made those of us in basic sciences deviate from our primary career goals.”

Furthermore, international collaborations were often required by the funder, restricting the local researcher from directly applying for a grant. For example, **Participant 7** wrote that s/he “*decided to collaborate with an international partner because it was a grant requirement that researchers from resource-limited countries should partner with researchers from developed countries*.” Likewise, **Participant 3** “*could not have qualified for the application unless I had a global (US-based) mentor.*”

Foreign dominance was also described in writing collaborative grant proposals. Some participants described a “*need for more collaboration on grant writing and submission*” while other jointly wrote proposals in which “*each member participated in writing sections of it*,” (**Participants 1 and 7**, respectively). However, **Participant 9** described a few different experiences in establishing foreign collaborations and the grant submission process,

“I have been engaged in several international projects which have had different approaches to the partnerships. There have been projects where we have drafted the concepts together and refined the concepts and crafted the methodologies together. In these projects we have had a common understanding and set common activities which have been costed for the budgets. These have been the most equitable collaborations… The resources have been shared equitably and I have held a subaward agreement. In those collaborations which have required a third world leadership, I have been the prime recipient with my international collaborators holding subawards. However, there have been some research projects which have been brought to us in Africa as comparative studies having been fully developed in the west. In these, we have had to conform to the dictates of the methodologies already worked out. However, we still have had the opportunity to refine the costing of the activities for the budget and have been allowed to pilot and sometimes vary the budgeting to allow for the local nuances.”

Her/His experiences demonstrated variation in research collaborations, which was found across participants. This variation, especially within one section of a journal entry, presented challenges in interpreting experiences. Phrases of this excerpt were coded as effective collaboration as well as research colonialism and represent the complexities in these collaborations.

There was an imbalance of leadership at the macro-level. **Participant 2** noted that “*It is also important to balance the power between the national and international collaborators on a research project, including opportunity for leadership roles*.” **Participant 4** desired greater leadership, “*While we are glad with the current state of collaboration with non-in-country collaborators, I would be glad to play more senior roles such as Multi-PI status*.” Additionally, participants wrote about the leadership related to budgetary power that foreign partners held. **Participant 10** noted, “*budget lead is not possible for local leadership*.” S/He further explained that “*if we as a group decide to take up such research then all the final budget decisions are made in the international office*,” not locally where the research is being conducted as one might expect.

Participants also described that researchers from LMICs encounter negative bias from international journals that are predominantly located in high-income countries. **Participant 3** explained that,

“The good journals are not immune to some bias and prejudices. You are automatically disadvantaged if you are writing from a low-income country with no pedigree for good research. You need to be extraordinarily patient, because you will get a lot of automatic rejections from good journals. This is mostly because someone somewhere assumes that nothing good will come out of such a place that has no pedigree, particularly if the work is not associated with renowned partners in Europe and America. It is certainly hard for a young academic, but it is a reality he or she must face while growing up.”

Regarding co-authorship with foreign partners, participants wrote about opportunities to publish with their foreign collaborators. Further describing these experiences, **Participant 9** reflected that “*some collaborations have required prior signed agreements while in some we have had to agree on these on an ad-hoc basis*” and that they “*advance on the principles of getting full appreciation of the activity and getting the most appropriate representative to take leadership*.”

Despite power imbalances and challenges, about half of the participants used positive words and phrases when writing about international collaborations. They described their experiences as “*a healthy one*” **(Participant 1)**, “*amazing*” **(Participant 3)**, “*interesting*” **(Participant 5)**, “*exceptional*” **(Participant 9)**, and “*good*” **(Participant 6)**. **Participants 3 and 9**, respectively, wrote, “*There is nothing I would have wished to change in my experience of working with my international partners*” and “*In all, my experiences have been quite positive and I’ve enjoyed every moment of my engagement on international collaborative research projects.*” Furthermore, participants wrote that these research relationships were “*mutually beneficial*” and “*helped achieve results faster and better*” because of the “*wider skills and vast array of resources available for the project*” (**Participants 3, 1, and 9**, respectively).

### 3.2 Finding 2—microsystem: local challenges

All participants, regardless of their number of years in research or their position, reflected on experiences of institutional and national resource challenges within their microsystem. These challenges were often complex and multi-faceted. In establishing a research career, participants faced obstacles related to time, infrastructure, funding, and mentorship. Participants wrote about the ways that these resource limitations hindered their research productivity and hence, their scholarly growth and professional autonomy. However, in their reflections about these challenges, they demonstrated resiliency. To overcome these challenges, participants described turning to foreign collaborations to fund their research, obtain resources, and find mentorship.

Having adequate time to conduct research activities was a major challenge to many participants. **Participant 2** reflected, “*it was hard to dedicate time and resources to a research career*” because “*we have to work in multiple settings, in order to make ends meet… it was quite difficult to dedicate time to research*.” For example, **Participant 1** explained, “*clinical work is so voluminous that it eats into the time left for research*” but that participant *“tried to overcome this.*” The lack of time often resulted in “*some activities or assignments were not completed as quickly as was expected by other international colleagues*” (**Participant 5**). However, experiences with foreign collaborators gave her confidence to negotiate with her institution for protected research time,

“Experience from these collaborations has given me more confidence in my academic writing and also in negotiating with my employers, especially with regards to protected time for conducting research, which in most low-and middle-income countries is very difficult to get.”

Regarding the local systems, almost all participants wrote about a lack of funding for research, salaries, and journal publication fees. **Participant 1** reflected, “*after overcoming time constraints, is the challenge of funding for research activities*.” **Participant 3** described the “*disturbing*” reality that “*researchers rarely find any funding support from the government or non-governmental organizations*” in their countries. Continuing, s/he added that “*the salary is very low compared to practitioners who chose to work outside the academic circle*.” **Participant 7** attempted to explain the funding situation and research implications: “*institutions in African settings have little stimulus packages to motivate development of research studies locally*” resulting in “*the full spectrum of ideas one would want to explore has to be limited for cost considerations. This approach affects the quality of research and limits the creativity expected from a researcher*.” S/He described “*overcoming these challenges through doing basic research that does not require huge resources and by collaborating with international institutions*.”

Participants also used personal funds to pay for manuscript publication fees but that “*greatly affected the caliber of journals… because some were too expensive*” **(Participant 5)**. **Participant 2** noted, “*publication fees are sometimes too high*,” which was exemplified by **Participant 3** as “*higher than the average monthly earning of an academic from our country*.” To address this challenge, some participants requested for waivers from high impact journals, highlighted by **Participant 6**: “*we cannot arrange the money as this particular study was an unfunded activity and we did it because of our scientific rigor and for the overall benefit of TB/HIV prevention in* [country].”

Funding also affected **Participant 2**’s ability to pursue research training because s/he “*could not afford paying online course fees.*” S/He also wrote about the high fees for “*participating in scientific conferences*” and has found that “*moving to a research institution allowed me to have access to some (few) training tools*.”

Several participants noted challenges related to infrastructure as they worked to conduct research independently and with foreign teams. **Participant 9** described, “*the largest handicap I face is no official Grants Office at my institution*.” **Participant 7** also noted the need for the “*strengthening of the grant office.*” Additionally, he wrote that “*institutional ethics committees are not streamlined in most resource-limited countries*,” which was further described by **Participant 2** as “*very bureaucratic processes in the country for study approval by an IRB that can take almost a year.*”

A “*big barrier*” that many participants faced was a “*lack of academic mentorship*” (**Participants 2 and 5**, respectively). As described by **Participant 5**, “*until very recently, this was the norm in most* [country] *institutions*” and s/he “*never had anyone mentor me on academic writing until I went for the* [foreign training program].” This lack could be explained by **Participant 7**’s observation that “*This guidance is largely missing in African settings due to insufficient mentors*.” **Participant 2** explained that, like the participants, “*Mentors and supervisors are themselves involved in multiple tasks and activities that little time is left for mentorship. It may take months to receive the feedback from a work submitted to them for review*,” thus delaying research and academic progress.

Some participants sought guidance from foreign mentors and collaborators, while other participants developed workshops and programs to build a pipeline of local mentors. For example, **Participant 4** recently submitted a grant application to develop and provide “short-and *medium-term learning opportunities, paired mentoring arrangements and seed funds to eligible mentor-mentee teams*” to “*create the next generation*” of clinical researchers.

### 3.3 Finding 3—mesosystem: dependency

Many participants overcame local challenges through foreign collaborations that provided funding. Their descriptions revealed dependence as they relied on requests for collaboration that would provide research funding and other resources that were needed to move research projects forward. Through this dependence, they created a mesosystem that between the local microsystem and international macrosystem.

**Participant 6** reflected, “*I believe if we want to excel in research then we have to establish foreign collaborations. This is particularly true for researchers from developing countries*.” For some, collaborations filled a technical gap, as described by **Participant 9**, “*The proposal required a complement of technical expertise, which was inadequate at my institution*.” **Participant 5** attributed success to “*collaboration with people outside my country that have the experience of writing successful grants and mentoring.*”

Participants depended on their collaborators to send information about grant opportunities: “*publicity of these calls for proposals was done only to exclusive audiences and thus we had to depend on our colleagues in the west to see them and send them to us*” (**Participant 9**). Due to such dependence, **Participant 9** commented, “*I have not established an independent research career and I do not think I ever will*,” despite having co-led multiple grants.

**Participant 9** explained that s/he looks abroad for funding because there is “*very little local funding for research which makes it difficult to pursue local research priorities thus keeping our eyes focused more on global health research*.” Similarly, to overcome local funding challenges, **Participant 5** described, “*I made up my mind to start writing grants so that I can access some of these funds to conduct research in* [home country]*, where there is so much to do and no resources*.” Using foreign collaborations to fill gaps in infrastructure and expertise was “*necessitated*” (**Participant 7**) to continue their research. **Participant 10** wrote that in working with an international group, “*we have established a research office in my country*,” which “*has helped me remain in research*.” Similarly, **Participant 3** described funding research through foreign collaborations,

“The opportunity I got from working with international mentors paved the way for me to understand how to access extramural grants and fellowships from international organizations to support my modest research ideas. This is one of the biggest motivators.”

However, in seeking international funding, **Participant 9** worked to move away from dependence by strengthening capacity within his institution so that they could bring equity to research by leading grants rather than being dependent on foreign leadership. S/He described,

“I have tried to minimize these disadvantages by training a grants management team which would understand research compliance processes and be linked into the research information systems so as to adequately advise on any calls [for grant proposals] being planned for and also on identifying appropriate partners. As we build this capacity, we prepare ourselves to prime [i.e., submit] on some of the grants and invite our partners to collaborate. This gives a sense of equity in the research arena.”

### 3.4 Finding 4—scholarly identity

In their reflections, participants described relatedness within collaborations during impactful experiences in foreign training programs and through ongoing mentorship. In foreign collaborations and trainings, participants experienced recognition as a scholar, especially when they published and presented their research. Interpreting these reflections, the experiences motivated them and enhanced their confidence and identity as an international researcher. In addition to this extrinsic motivation to research, participants expressed intrinsic motivations. They reflected on how their research had the potential to and did generate evidence for policy change to improve health outcomes.

The scholarly and career impacts of formal training experiences were central points of reflection for some participants. **Participant 5** described, “*during this period* [of grant writing training in the USA]*, I was introduced to ‘proper’ academic writing*… *by the end of* [training program]*, my first manuscript as a lead author in a high-impact journal was almost ready for submission!*” In a similar vein, **Participant 2** reflected on “*having not much experience in grant writing and submission*” and noted that “*recent experience in* [grant writing training program] *was very important for my research career*.”

Reflections of skill development, increased competency in grantsmanship, and career growth were interwoven with descriptions of collaborations and mentorship. **Participant 7** summarized, the “*research career path development requires guidance from those who are established*,” highlighting the impact of mentorship in research and professional development. Diving deeper, **Participant 6** described mentorship as a formative process, “*During this whole process I was not spoon fed. I was guided, made to think, provided literature that I had missed so that I could develop the research proposal myself, but under the supervision of my mentors*.”

Several participants described joint publications with their international mentors as well as the formation they received in the process. **Participant 5** described,

“At this point in my academic career, I am glad to say that my international collaborations have changed my outlook to academic writing completely. I have leveraged on the knowledge I have gained and improved on my academic writing, now I am better placed to develop and submit quality academic writing with the support of my mentor.”

Participants also described publishing as lead author with their mentors including, they “*gave me the opportunity to be the lead author in that publication*” (**Participant 5**) and “*I have had equal opportunity to be first author on some of the publications from collaborative works*” (**Participant 9**).

Some participants explained that publishing and researching to generate evidence for change motivated them. **Participant 1** was encouraged by “*seeing the results of our research published in academic journals and being cited.*” Others found scholarly identity in the recognition that came with publication. After **Participant 3** experienced her/his “*first breakthrough of publishing in an international journal through that collaboration*,” s/he described receiving “*visibility in the sense that now some of the international journals send articles for me to review*.” Additionally, s/he expressed pride in being asked by a foreign collaborator to partner on a new grant, describing it as,

“After delivering the first project successfully, my collaborators appreciated my methods of managing our local team. This fetched me another collaborative project with them. My team maintained a similar approach. We worked harder and brought on our experience of the previous work to bear.”

In that and other ways, participants described becoming more confident in their scholarly identity through foreign collaborations. **Participant 9** explained, “*This interaction with other scientists in different jurisdictions is very satisfying to a researcher and gives one a sense of self-worth that is incomparable*.” Additionally, **Participant 10** described that foreign collaborations led to “*recognition within the country for contribution to the field*” as well as “*recognition in country and internationally so that research groups have approached me as an individual to conduct their research*.”

However, participants’ scholarly identity was not simply defined and motivated by extrinsic recognition. They described intrinsic motivation to pursue scientific discovery that would change policies and improve health outcomes. As **Participant 10** described,

“Though I came into research kind of by accident I have remained in it because it provides an avenue to participate in generating evidence for health. As I practice medicine, I see how research changes practices and stimulates more questions. The recognition that comes with being part of impactful research is great but also the lessons and further questions that arise from negative studies is important for further research.”

Through their research, participants engaged with local government and hospital leaders. **Participant 9** reflected, “*the linkage of research with civil society work has been an exciting side*.” Participating in these conversations “*provided a wider platform from which to disseminate the research findings to an audience that is keen for knowledge translation mostly aimed at evidence-based policy formulation and/or implementation*.” **Participant 5** worked with “*the government to key into our program to ensure sustainability of the project*.” S/He further described how one of her grant-funded studies “*completely changed the management of children with sickle cell disease… in our hospital and state as a whole*.”

### 3.5 Finding 5—leadership

Participants were leaders within their local institutional teams. They described leadership with emphasis on the group’s well-being and collective decision-making. However, their reflections revealed a disparity in leadership dynamics while collaborating with their foreign partners. While actively contributing to the team, participants perceived a lack of leadership in the international macrosystem and expressed a desire for more substantial leadership roles and recognition.

Participants overwhelmingly described leadership as a group effort, summarized concisely by **Participant 10**: “*obviously leadership is seen in ability to function in a team*.” In describing their leadership, many participants wrote about regular, *“weekly and sometimes fortnightly*” team meetings in which “*from conception to execution, decisions were taken collectively*” (**Participants 1 and 3**, respectively). In general, participants noted that decisions “*are taken collectively*” and “*were made by discussions with the whole team*” (**Participants 5 and 1**, respectively).

In leading local teams, **Participant 3** “*made them understand the project is not all about me. It is about all of us, and nothing should stop because I or any one of us is not around*.” In this fashion, weekly team meetings, which were initiated “*to make everybody feel onboard… including the coordinators*,” continued to occur “*even when I traveled*.” Furthermore, decisions to accept a project proposal were taken together because “*whether we should go ahead or decline is based on our collective interest, not my interest alone*.” Similarly, **Participant 5** described, “*I will always bring issues to the table for discussion and I hear everybody’s suggestion. We critically discuss and analyze all the suggestions and I eventually use what I have learnt from the team to make the final decision*.”

**Participant 10** described leading the local team as “*decisions would be made centrally but it was expected that as a local leader, customizing for the local context was up to the leader.*” However, s/he described challenges with being recognized as a leader in collaborative grants, further explaining,

“Another critical role is to act at the interface of the organization and external stakeholders. Through the years, the duplication of leadership roles I believe is a challenge that one working in collaborative research environments needs to be aware of. It becomes easier to negotiate with higher responsibility but there seems to be someone else trying to do your job.”

Explaining his experiences further, **Participant 10** described,

“I am included as an investigator in many grants that come in but not necessarily pushed to do on my own… What I have noted in these positions, due to the need to have a link with universities abroad there is always a feeling that I am not really the lead. Obviously, leadership is seen in the ability to function in a team but the dual structure of the local office and the abroad structure make it difficult to lead, particularly team members who seem to answer only to the abroad component of the structure.”

However, s/he later noted that, “*With time, my role evolved with taking up roles of investigator and my leadership role also became more ‘real’*.”

Most participants also described leading only at the local level, although they still experienced a collaborative effort within the larger foreign team. **Participant 4** explained, “I have been on the know from drafting the grant application through submission and subsequent award. I am comfortable with the level of transparency and collaborations.”

Similarly, **Participant 7** described collaboration as well as his input on team discussions,

“Most decisions were made during the meeting times. We discussed issues and, by way of consensus building, we arrived at what was to be done. Since I knew what we needed to achieve at the end of the project, I always provided insights for deliberation and so it was easy to agree.”

However, reflecting on a lack of personal leadership in the foreign team, **Participant 7** suggested that “*participants from resource-limited countries lead the projects as principal investigators.*” S/He explained that s/he has “*endeavored to identify grant opportunities myself and then request international partners to come on board. This is in an effort to be the PI of the project*.”

### 3.6 Finding 6—foreign perspective

In their reflections, participants seemed to adopt their collaborators’ perspectives on various topics such as timeframes and project management. However, they expressed a desire for changes in their collaborators’ viewpoints, particularly in leadership and budget considerations, to better align with the local context of the research projects. Additionally, participants reflected that often collaborators held unrealistic expectations for task completion times due to a lack of understanding of local infrastructure limitations.

Participants and their collaborators often had differing perspectives on reasonable timeframes for project tasks that appeared to stem from foreign collaborators’ lack of understanding about local systems and processes. **Participant 2** summarized this lack of understanding:

“It is also very important for international partners to bring more realistic timelines as the IRB processes in the host country may differ and take longer than in their countries. Field implementation also has many challenges such as: delays, difficult access to roads, transportation, the need for local authorities to authorize the data collection, etc. Things move on a paper-based approach here rather than the internet, meaning that these processes take a lot of time. There was an unreasonable pressure to move things on tight deadlines set by international partners. In the end, there was an understanding but it took time to reach that. Overall, international partners need to be more aware of the realities in the countries they propose for research collaboration or at least be willing to hear from their collaborators.”

This participant was adamant about the need for collaborators to understand the perspective of local researchers, particularly related to time needed to achieve elements of the research process. **Participant 1** had similar reflections, although seemed more forgiving of the collaborators’ lack of perspective:

“Collaboration with people from the States has helped achieve results faster and better. This international experience is a healthy one. The only problem is that sometimes there is pressure to ensure all deliverables are done on time. If this pressure can be reduced sometimes it will be appreciated. This experience with collaboration has helped influence my approach that you do not play with deadlines and that you have to have a communication plan and communicate effectively all the time.”

As an example of the benefit of aligning expectations, **Participant 9**, who had “*exceptional*” experiences in international collaborations, felt “*my responsibilities have been clearly laid down and I have had the required respect that demands the performance of my responsibilities*.” Similarly, **Participant 3** reflected that,

“My experience of working with my international partners made me believe that working with other partners would be seamless and hitch-free. I am now confident to work in a team on grant writing, while clearly agreeing on each other’s responsibility and resource sharing formula from the beginning.”

Some participants struggled with foreign collaborators to develop clear understandings of leadership roles and team collaboration. For **Participant 6**, “*Our relationship is good although sometimes I feel that one partner, a senior researcher, tries to impose his rules and ideas to junior researchers, such as myself, without discussing first with all members of the team*.”

Later in the journal, **Participant 6** reflected on being mentored by a foreign partner and noted their different perspectives for professional relationships:

“My mentors would give me plenty of time to talk and discuss about the research work, but generally we did not discuss much about our personal lives. So, at times the meetings were too professional. I was not encouraged too much to talk about my issues other than the research topic. I particularly wanted their guidance regarding my future career development but maybe because of their busy schedule and other commitments they could not provide me guidance related to career development and the opportunities available in the public health field.”

## 4 Discussion

Interpreting the six findings with the frameworks (Self-determination Theory and Complex Systems Leadership Theory) and the literature review, we infer that participants were, (1) motivated by scholarly identity in collaborations, (2) depended on collaborations that were colonialist but beneficial, and (3) created change through leadership at the microsystem level (Table 3).

Similar to the literature (e.g., Färnman et al., 2016), participants participated in foreign training programs to strengthen their grant and academic writing and described positive impacts on their scholarly identity that stemmed from mentorship in these training programs and other foreign collaborations (Finding 4). Authors in the literature reported that researchers increased their ability to secure grant funding after foreign training. However, participants described desires for greater leadership and budgetary control, suggesting that they were not securing their own grants (Finding 5).

Our findings aligned with descriptions of research colonialism in the academic literature (Tan-Torres Edejer, 1999; Chu et al., 2014) in which foreign researchers led the research and provided connections for funding (Finding 1). Similar to the literature, participants described leadership imbalances and being approached by foreign researchers with studies that were conceptualized elsewhere (Kok et al., 2017; Ward et al., 2017; Seo et al., 2020). However, contrary to the literature, participants noted being included in grant proposal development and almost half positively described their experiences in foreign research collaborations. Nevertheless, participants’ descriptions of differing perspectives on deadlines and leadership roles (Finding 6) and the need to clearly define responsibilities at the outset of the grant (Finding 2) aligned with the literature (Nchinda, 2002; Vasquez et al., 2013; Munung et al., 2017).

All participants described their experiences of personal, institutional, and national challenges to establishing an independent, productive research career (Finding 2). Participants’ descriptions of local challenges in funding, infrastructure, time, and mentorship were almost identical to the literature (e.g., Ager and Zarowsky, 2015; Kumwenda et al., 2017). Their descriptions of collaborating with foreign researchers to fill local funding gaps, as well as associated challenges of power imbalances, needing to align with funders’ agendas, and a lack of agency, echoed the literature (Walsh et al., 2016; Harsh et al., 2018). Missing from the literature were the ways that productivity and careers were stagnated by biases and power dominance (i.e., research colonialism).

Many participants led local research teams and their descriptions of leadership aligned with elements of collective leadership (Finding 5) that were described in the literature (Denis et al., 2017; Edwards and Bolden, 2022). Participants generally described leadership as a group effort and through leadership, changed processes and policies for improved health outcomes.

### 4.1 Inference 1—motivated by scholarly identity found in collaborations

Through foreign collaborations, participants grew their scholarly identity, which motivated them to continue researching. Participants received mentorship, which instilled feelings of relatedness with the global research community and led to collaborations, publications, and conference presentations, which increased their feelings of competence and self-efficacy.

Extrinsically, participants were motivated by recognition from publication and engagement on an international stage. Intrinsically, participants were motivated by their research contributing to the evidence base for changes in healthcare policies. Locally, they influenced leaders who changed clinic protocols and testing procedures. Participants were invited to national and international policy discussions in which they shaped policy change.

**FIGURE 1:**
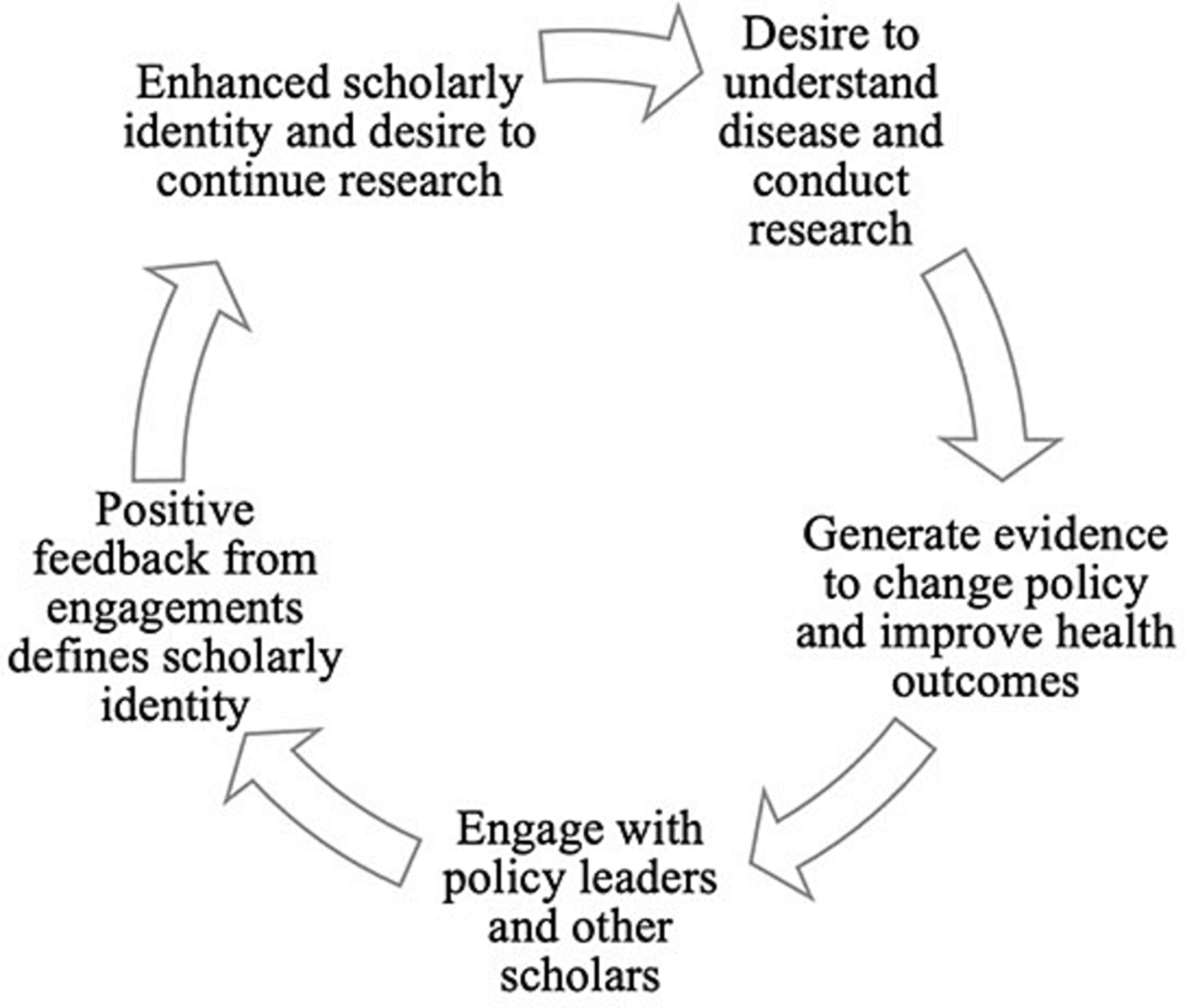
Feedback loop: motivation, research, scholarly identity, and foreign collaborations.

Relationships were foundational elements of participants’ motivations and experiences in foreign collaborative research. Collaborations provided pathways to improve health outcomes as well as positive feedback and recognition, which motivated participants in their research aspirations. Participants’ motivations to research moved them to remain engaged in foreign collaborations to fund their research endeavors, which further fueled their research motivations. Participants’ engagements in collaboration and research shaped their scholarly identity and created a positive feedback loop that motivated their investment in research (Figure 1).

### 4.2 Inference 2—depended on collaborations that were colonialist but beneficial

**FIGURE 2:**
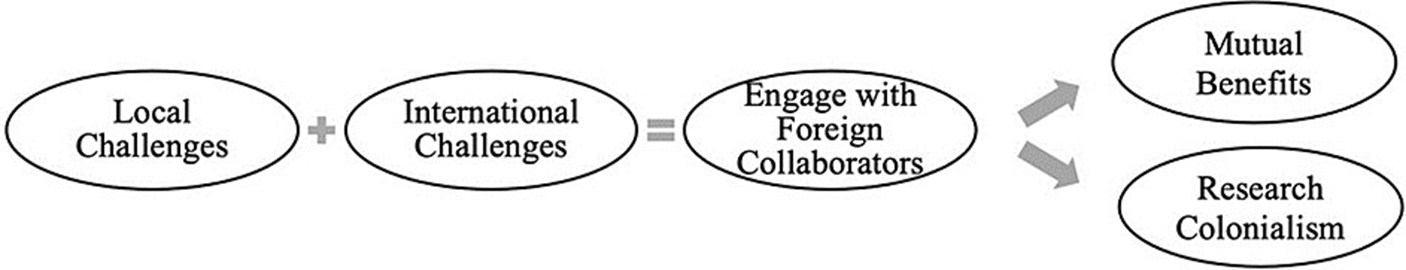
Navigating challenges through foreign collaborations (colonialist but beneficial).

Faced with limited local research funding and infrastructure as well as discriminatory biases in international academic spaces (e.g., journals and grant funding), participants used foreign collaborations to address these challenges and support their research endeavors. However, in doing so, participants experienced overt and subtle foreign dominance and power imbalances (Figure 2). These experiences were pervasive throughout the research process and characteristic of research colonialism described by authors in the academic literature.

A colonialist environment was evident at the international macrosystem level through biases against participants when they tried to publish in international academic journals without foreign collaborators and in the high costs associated with publishing research articles, international conference registration, and international training. Furthermore, despite being outside the community being researched, foreign funders often controlled the research agenda and required that local training be led by foreign researchers rather than local experts. These macrosystem challenges added to the microsystem challenges that participants encountered.

Despite challenges at the international level, participants leveraged foreign collaborations to navigate through local challenges, creating a mesosystem between the local and international systems. Participants used grant funding from collaborations to conduct studies, publish articles, and attend training programs. Through training programs and mentorship, they described strengthening their skills in grantsmanship and academic writing. With increased competencies, they were more confident in applying for grants and making change in their institutions.

However, in an environment of research colonialism, participants depended on foreign researchers to approach them to initiate collaboration. In this dependency, participants lacked autonomy and were often disempowered to fully lead grants. Foreign researchers were empowered by funding mechanisms that prioritized them over local researchers as grant leaders. Once engaged in foreign collaborations, participants also experienced power imbalances through funders’ preferences of foreign collaborators to control budgets and lead training. This view permeated through the research team, sometimes influencing local team members to favor the foreign grant leader.

### 4.3 Inference 3—created change through leadership at microsystem level

Through adaptive interactive behaviors, participants demonstrated leadership within complex systems and led local teams. When challenges arose, participants adapted through linking with foreign collaborators. Complex Systems Leadership Theory proposes that to overcome challenges in a system, individuals should link up to develop adaptive behaviors that will become superior to previous actions. In navigating challenges and influenced by their foreign collaborators’ perspectives, participants demonstrated leadership and changed norms in their spheres. Whether through asking a journal to waive publication fees, negotiating for research time, or building a local grant administrative team, participants’ actions created new local precedents. Furthermore, participants used collaborative research to solve problems related to disease and community health. Through linking with others, participants provided evidence for new policies to improve health outcomes.

However, despite leading their local research teams and emerging as local and national leaders, they lacked substantial leadership in the foreign team due to an environment of research colonialism. As they mitigated local challenges through foreign collaborations, they often encountered new challenges (i.e., power imbalances). Participants discussed ways that they strived for increased leadership and autonomy in their collaborations, and some demonstrated that they were beginning to make changes. Their motivations to research pushed them to link with foreign collaborators and subsequently enhanced their competence and strengthened their desire for increased leadership. Through sustained engagement, it could be presumed that participants continually embark on larger change and increased leadership, which may decrease the power of foreign dominance and research colonialism over time.

### 4.4 Strengths

Our research participants represented a diversity of countries, backgrounds, and research interests (see Table 1), which helps generalize findings to a larger population. Additionally, because they were currently involved in research and have been for a range of years (at least 2–15), experiences may have spanned over a decade and shed light on long-term as well as recent issues in international collaborations. The use of journals provided participants time to reflect on questions and respond in a thoughtful manner. Furthermore, the tool also created distance between researchers and participants and allowed participants to remain anonymous, which may have increased participants’ honesty in their answers.

### 4.5 Limitations

There were several limitations in this study. The participants involved in the study had been trained at the lead author’s institution and participants may have been hesitant to share demeaning experiences. Journals did not permit follow up (“probing”) questions after submission, which could have been used to gather additional descriptions about an experience. Furthermore, the written reflections did not capture body language nor voice intonation that may have aided in the interpretations of responses. Finally, we did not seek to understand the impact of variations in cultural and regional perspectives, and these variations may have impacted how participants interpreted experiences.

### 4.6 Recommendations

Based on the findings and inferences, we recommend five actions for international collaborative research and future research directions in Africa (Table 4).

## 5 Conclusion

Our study shows the important role of foreign collaborations for researchers in Africa as partnerships enhance their scholarly identity and help them overcome challenges in funding and mentorship. At the same time, this research linked study participants’ engagement in foreign collaborative research with research colonialism. The participants experienced overt and subtle research colonialism as there were power imbalances at all stages of grant and research processes, even when the participants reported being satisfied with the collaboration. A major concern is the foreign collaborators’ lack of understanding of issues pertaining to time constraints and infrastructure deficiencies in the local setting. Strengthening systems leadership in research through training and mentorship could continue to increase self-efficacy and competency of African researchers. Paired with greater awareness of and action against bias and power dominance by foreign funders and collaborators, the presence of research colonialism in academic biomedical research might decrease and give space for local, African researchers to lead grants in their communities.

## Supplementary Material

Supplementary materials - PDF version

Supplementary material

The Supplementary material for this article can be found online at: https://www.frontiersin.org/articles/10.3389/feduc.2023.1217066/full#supplementary-material

## Figures and Tables

**TABLE 1:** Demographics and attributes of participants (*n* = 10).

Category	*N* (%)
Country	
Ghana	1 (10%)
Mozambique	1 (10%)
Nigeria	3 (30%)
Zambia	4 (40%)
Did not disclose	1 (10%)
Gender	
Female	3 (30%)
Male	6 (60%)
Did not disclose	1 (10%)
Years in international research	
2	2 (20%)
3	1 (10%)
4	1 (10%)
5	3 (30%)
7	1 (10%)
12	1 (10%)
15	1 (10%)
Research area (many participants wrote multiple areas, hence total is greater than 10)	
Cardiovascular science	1
Epidemiology	2
Health equity	1
Health systems	2
Hematology	1
HIV/AIDS	1
Implementation science	1
Infectious diseases	1
Maternal health	1
Microbiology	1
Pharmacology	1
Primary care	1
Sickle cell disease	2
Did not disclose	2

**TABLE 2:** Findings with exemplar quotes from participants.

Findings	Exemplar quotes from participants
Macrosystem: foreign dominance	• The decision to apply was at the instance of the [foreign institution] team. (Participant 4) • This makes us remain as sub-awardees sometimes to grants that are targeted at Africa and reduce access to funds in that the prime [i.e., foreign institution] takes a larger portion of the funds (Participant 9) • The collaboration was mutually beneficial considering that our international partners have access and skills needed to acquire the funding, while we can support the project with efficient recruitment. We needed each other to answer, convincingly, the research question we have. (Participant 3)
Microsystem: local challenges	• Another huge challenge is creating time to write. Our academic system does not give ‘protected time’ for research especially for junior faculty thereby, limiting their ability to generate preliminary data for applying for grants. Therefore, for any sort of academic writing, we had to bend over backwards! (Participant 5) • You could imagine spending more than your monthly earnings to publish in a good journal. Definitely, you cannot publish much due to the cost. Sadly, the local journals that are affordable have no rating and many of them are not indexed. (Participant 3)
Mesosystem: dependence	• To overcome this, I reached out to my old colleagues and tried to conduct research and get these published. (Participant 6)
Scholarly identity	• Collaborative research has also strengthened the other members of the team to the extent that the skills we gained has made us stand out in our institution and a number of collaborators have reached out to us on the conduct of some studies. (Participant 5) • Though I came into research kind of by accident, I have remained in it because it provides an avenue to participate in generating evidence for health. As I practice medicine, I see how research changes practices and stimulates more questions. The recognition that comes with being part of impactful research is great but also the lessons and further questions that arise from negative studies are important for further research. (Participant 10)
Leadership	• There were times when challenges on patient management were brought to the forum and such issues would be discussed thoroughly until a general consensus was met. Decisions are often taken collectively with the guidance from both the protocol and the local ethics. (Participant 5)
Foreign perspective	• The most challenging thing about this experience was the terms and conditions for this collaboration, around data sharing and payment for the national team members, considering that an amount of work will be done in the country. I have learned that aspects of data sharing, authorship and financial aspects are very important and to be discussed upfront. (Participant 2) • There was also some level of negotiation within the organizational structure to clarify what my colleagues expected my role to be. It seems that the structure would have duplicate leadership roles so that it required documentation of what my roles were so that we knew what to expect. (Participant 10)

**TABLE 3:** Inferences with supporting elements (i.e., findings and frameworks).

Inferences	*Supporting elements*	
	Findings	Frameworks
Motivated by scholarly identity in collaborations *Participants enhanced their scholarly identity through foreign collaborations, which contributed to their motivations to remain engaged in research.*	Microsystem, scholarly identity	Self-determination Theory (competence, relatedness, motivation)
Depended on collaborations that were colonialist but beneficial *Participants depended on foreign collaborations for research support but encountered research colonialism.*	Macrosystem, mesosystem, foreign perspective	Self-determination Theory (autonomy) Complex Systems Leadership Theory (Unpredictability, Emergence)
Created change through leadership at local levels *Participants led at microsystem levels with a focus on collective decisions and they created change as they emerged as leaders in challenges.*	Microsystem, leadership	Complex Systems Leadership Theory (unpredictability, emergence, leadership)

**TABLE 4:** Recommendations for practice based on the study inferences.

Recommendations	Rationale
Encourage bidirectional training and mentoring opportunities	Increase feelings of relatedness that motivates continued research as well as collaborators’ investment (Inference 1)
Require participation in power assessment and training as prerequisites for international grant funding	Decrease foreign dominance and subtle research colonialism in collaborations (Inference 2)
Prioritize local leadership and perspective in research grants	Decrease dependency on foreign collaborators (Inference 2) and increase opportunities for grant and budget leadership (Inference 3)
Review leadership responsibilities and leverage collaborative leadership	Enhance opportunities for leadership and leverage collaborative leadership that leads to change through challenges (Inference 3)
Remove publication barriers (e.g., reduce or waive publication fee) for researchers in LMICs	Increase scholarly recognition and identity for researchers (Inference 1) and diminish research colonialism (Inference 2)

## Data Availability

The original contributions presented in the study are included in the article/Supplementary material, further inquiries can be directed to the corresponding author.
