# Supplementary material for: International collaborative research, systems leadership and education: reflections from academic biomedical researchers in Africa
Source: Front Educ (Lausanne). Author manuscript; Available in PMC 2026 Jun 27. (PMC13309179; doi:10.3389/feduc.2023.1217066)
Supplement: Supplementary materials - PDF version [file NIHMS2173074-supplement-Supplementary_materials_-_PDF_version.pdf]

*Supplementary Material*

# International Collaborative Research, Leadership and Education: Reflections from Academic Biomedical Researchers in Africa

**Elizabeth S. Rose[1*], Halima Bello-Manga[2], Theodore Boafor[3,4], Muhammad Asaduzzaman[5*]**

*** Correspondence:**

Elizabeth S. Rose
elizabeth.rose@vumc.org

Muhammad Asaduzzaman

Muhammad.asaduzzaman@medisin.uio.no

## Appendix 1: Journal topics

### Topic 1 – International collaboration

Discuss your experiences with the distribution of resources, responsibilities, and outputs in international collaborative academic biomedical research projects. If you have participated in more than one project, please answer the questions taking into account all of your experiences. If you have only participated in one project, focus your answers on that one project.

Focus questions for additional guidance and thought:

- Please provide a story (or stories) to illustrate examples of the relationships between you and other team members. Please do not provide identifying information.
- Discuss the role(s) you played in various stages of the project. How did you feel about your role in the project(s)?
- How were decisions made in this project(s)? In what ways do you feel that you led or influenced team members and/or decisions? How do you feel about the way that the decisions were made?
- In your experience(s), what elements of collaboration were effective? What elements were ineffective?

**Topic 2 – Collaboration on Grant Writing and Submission**

Discuss at least one experience writing and submitting a grant with an international partner. Include elements particular to your research team, your institution's research system, and your national research system.

Focus questions for additional guidance and thought:

- Describe how and why you decided to collaborate with someone outside of your country.
- Describe what, if anything, you would change about this experience (or other experiences) with international partners.
- Describe the ways in which this experience (or other experiences) has influenced the ways that you approach grant writing and international collaborations.

**Topic 3 – Challenges in Establishing a Research Career**

There are a variety of challenges in building a research career in academic medicine. Related to your research career, discuss some of the challenges that you have faced at your institution (or at a previous institution), at the national level in your country, and at the international level. Provide stories or examples to illustrate these challenges.

Focus questions for additional guidance and thought:

- To establish your research career, you have demonstrated a high level of self-determination to overcome many of these challenges. Discuss intrinsic (internal) and/or extrinsic (external) motivators that have kept you on a research career path.
- If you feel that you have established an independent research career, please explain the ways in which you have established independence and authority over your projects as well as challenges you may have faced and overcome along the way.
- If you do not feel that you have established an independent research career, please explain the barriers in establishing independence, how you have tried to overcome these challenges, and the outcomes.

**Topic 4 – Your Leadership Behaviors and Roles**

There are many ways that members of a group lead. Some have formal leadership roles, such as a PI, while others informally lead through leadership behaviors influencing group decisions or ensuring that high quality work is completed on time. Describe the ways in which you have demonstrated leadership behaviors or assumed leadership roles that guided or influenced work on international collaborative grant-funded projects.

Focus questions for additional guidance and thought:

- Please provide a story (or stories) to illustrate examples of your leadership roles and behaviors.
- Please provide a story (or stories) to illustrate examples to describe your level of influence in decision making in your international research projects.
- Describe your leadership over the years through different projects and collaborators.
